# A Case of Abdomino-Intestinal Wound, Successfully Treated

**Published:** 1844-08

**Authors:** Andrew R. Kilpatrick

**Affiliations:** Woodville, Mississippi


Art. II.—*A Case of Abdomino-intestinal Wound, successfully treated.* By Andrew R. Kilpatrick, M. D., of Woodville, Miss.

The following case occurred on the Bayou Huffpower, Avoyelles Parish, La., in the year 1838:

Alfred, a negro man, æt. 47 years, while engaged 23d August, with a fellow servant in cutting down a large tree, both standing facing each other, the other man's axe flew off the helve in the downward stroke, and with considerable force entered the abdomen of the patient, dividing the recti abdominal muscles transversely, at the line dividing the left iliac from the left lumbar region, more than six inches in extent; and also dividing the ileum transversely fully eight lines. The whole blade of the axe entered and it did not fall out till his clothing was removed, when it was followed by nearly a hat-crown full of the descending colon, ileum and jejunum, which were loaded with alimentary substances, melons and seeds. The accident happened at half past 4 o'clock, P. M.; it was an hour and a half before I saw him, at which time he was nearly exhausted with loss of blood and pain. Some stercoraceous matter, shreds and seeds of melons were passing constantly from the orifice. The intestinal coats and mesentery were highly injected with blood, and there was a dark areola around the wound in the ileum. No part of the omentum was exposed, or visible.

Owing to the gorged state of the entire alimentary canal, I had considerable difficulty in returning the protruded portion; but after squeezing out some of the contents they passed in more readily than was at first expected. I was particular in retaining the wound of the intestine at the external orifice during the manipulations.

I now proceeded to the dressings of the wounds, and—

1st. Introduced a suture through the lips of the wounded intestine, brought the ends out and secured them externally, so as to keep the wound of the intestine at the abdominal wound to admit the escape of any discharge which might take place, and prevent, if possible, any from falling into the peritoneal cavity.

2d. Closed the external wound with three interrupted sutures, adhesive strips, compress and bandages.

At one time while returning the intestines his pulse was very slow and feeble, but at the time of applying the last dressings it was 65 and regular. He complained very much of the pain and soreness, comparing the sensation to that of a bar of iron of immense weight pressing on the abdomen. At 10 o'clock that night a dose of ol. ricini was given which was thrown back with a large amount of half-digested food of a disagreeable acid odor—other medicines were given, attended with the same results; the stomach retaining nothing but cold water.

24th. Rested well during the night. Slight febrile symptoms; no alvine discharge; urine passed with difficulty; no appetite; wound in good condition and discharging pus. At 8 o'clock, A. M., v. s. oz. viii; gave small dose of sulp. mag. which was retained with difficulty; applied large sinapism to epigastrium.

Evening. Two small evacuations obtained by glysters; pulse quick and frequent; v. s. oz. iv.; anorexia; abdomen tympanitic. Ordered small portions of sulp. mag. every half hour and enema at midnight if there should be no discharge before then; and v. s. if pulse warranted it.

25th. Morning. Rested well last night; two passages; pulse normal; less tympany; wound in good condition; adherent and granulating finely throughout its entire line; sutures all in *situ;* anorexia and nausea; applied vesicatory to epigastrium.

Evening. Three evacuations; much flatus escaped; bowels noisy and in constant motion; pulse 108, full, compressible.

Finding the suture in the intestine loose, I removed it very easily as it had cut through the coats of the intestine. There was no alteration produced in the wound by the withdrawal of this suture; adhesion progressed steadily. Still no appetite; tympany less.

26th. Morning. Rested well last night; one dejection during the night; pulse 80 and full; urinates without pain; took a little gruel.

Although I am averse to prognostics generally, yet I expressed the opinion that the patient would survive the injury.

27th. Morning. Rested well the 24 hours past; several copious alvine evacuations which contained melon seeds swallowed on the 23d. The wound was in a healthy state, granulating finely and rapidly; tympany almost disappeared.

28th. Evening. Continues to rest well; tongue slightly furred; pulse 68; borborigmy; appetite almost insatiable; adhesions seemed to be nearly thorough.

30th. Morning. Being otherwise engaged, I could not visit him on the 29th. Sleeps well, though weary of the recumbent posture. Felt some pain in the wound yesterday, which was followed and relieved by the discharge of much reddish-brown liquid from the orifice. Accidentally my pocket case of instruments fell on the patient's abdomen this morning which caused him to move suddenly and flinch, and immediately a vast quantity of fæcal matter, bile, bloody serum and pus gushed forth from the wound in a large stream. I made firm pressure on the abdomen and caused as much to be discharged as possible; air or gas also escaped in bubbles. The abdomen now became flaccid, and the lips of the wound were easily drawn in apposition and thus maintained by adhesive strips. Sutures having cut through were removed; appetite good; pulse 60. No passage on the 29th.

31st. Morning. Much pain in the sore; the above mentioned fluid constantly passed from it. On removing the dressings consistent fæces were seen lying in the wound. He observed that when he ate thin or watery diet it would escape from the wound in a short time, but if the food was solid it would pass on, stopping a little at the orifice as if hesitating whether or not to pass. The orifice is about three lines across; this was drawn together and secured tightly with numerous adhesive strips and the roller drawn as tight as could be borne.

Sept. 1st, 2d, and 3d. I determined not to meddle with the wound so frequently as formerly; for no doubt it retarded adhesion and sometimes tore up what had been imperfectly formed. Some watery discharge, but no bilious, or fæcal stain; repeated dejections, natural and easy; pulse 60.

5th. Found him much better, the wound having closed with the exception of a very small circular orifice, through which gas and a very slight portion of fæcal matter had passed.

7th. He now walks about without suffering any pain, and can sit up several hours in the day; opening smaller.

From this time to the 15th he rapidly regained his strength, and was able at this last date to hoe in the garden. Whenever any flatus collects in the bowels and moves down to the wound, it there stops a little, causing some uneasiness, but by pressing the hand over the part it quickly vanishes.

A furuncule formed under the cicatrix on the 12th, gradually maturated and bursted on the 20th, and discharged a quantity of pus. At this date the cicatrix is complete and much smaller, and neater than would have been supposed.

So this case was safe in less than twenty days from the time of the injury. The thermometer from 10 to 3 o'clock every day ranged from 87° to 93° Fah. He lay for more than an hour and a half with several feet of the alimentary canal exposed to the air, which was becoming dry from the exposure, and irritated by the constant handling of the negro men who were trying to replace it; he had to be transported, in a very inconvenient manner, nearly a mile to the house, and the thread through the intestine, and his own mental horrors in prospect of speedy death, were so many circumstances most unfavorable to his recovery. Yet he survived all, and was well and useful a few months ago. I am of opinion, from the circumstances and symptoms attending this case that the intestine has become attached to the peritoneal lining of the abdomen.

June, 1844.

